# Role of Endotracheal Tube Cuff Deflation in Facilitating Passage of Nasogastric Tube

**DOI:** 10.7759/cureus.28668

**Published:** 2022-09-01

**Authors:** Nidhi Bangarwa, Sumedha Vashishth, Vineet Kumar, Prashant Kumar, Preeti Gehlaut

**Affiliations:** 1 Anaesthesiology, Pandit Bhagwat Dayal Sharma Post Graduate Institute of Medical Sciences, Rohtak, IND

**Keywords:** abdominal surgery, general anaesthesia, cuff deflation, endotracheal tube, nasogastric tube

## Abstract

Background

Nasogastric tube insertion is a routinely performed technique in anesthetized and intubated patients. However, failure leads to repeated insertion attempts causing injury, which makes the situation cumbersome. Therefore, we introduce a simple technique of deflating endotracheal tube cuff for nasogastric tube insertion in such patients.

Methods

Eighty adult patients scheduled for elective abdominal surgeries were randomly allocated into two groups, Group A (nasogastric tube insertion without endotracheal cuff deflation) and Group B (nasogastric tube insertion with endotracheal tube cuff deflation) along with lateral pressure application at cricoid in both groups. The number of attempts required, overall success rate, the time taken, rescue maneuvers, and incidence of complications were compared between the groups.

Results

In Group B, the total success rate for nasogastric tube placement was significantly higher than in Group A (80% vs 55%, p=0.016). Group B had a 55% first attempt success rate, but only 25% of patients in Group A had a first attempt success rate (p=0.014). The overall time for successful nasogastric tube placement was longer in Group A than in Group B (65.4 ± 28.72 seconds vs 43.5 ± 25.37 seconds, p=0.0005). The differences in nasogastric tube kinking and coiling between Group B and Group A were not statistically significant (20% vs 22.5%, 10% vs 27.5%, p = 0.78 and 0.08, respectively). Mucosal bleeding was substantially higher in Group A than in Group B (30% vs 10%, p=0.025, respectively).

Conclusions

This study showed that endotracheal tube deflation significantly increased the first-attempt success rate, overall successful placement of the nasogastric tube in intubated patients, and incidence of complications like mucosal bleeding decreased.

## Introduction

The insertion of a nasogastric tube is an important procedure in abdominal, thoracic, and laparoscopic surgeries. It is a routinely performed procedure in intubated patients in the operating room. However, nasogastric tube insertion in an anesthetized and intubated patient is a tough job, with a high failure rate of around 50% on the first attempt using conventional technique [1]. In the conventional method, a nasogastric tube is placed blindly in the nasal cavity with the head in a neutral position. Failure leads to repeated insertion attempts causing trauma to oropharyngeal structures, which makes the situation more cumbersome. Furthermore, after every failed attempt at nasogastric tube placement, the incidence of hemodynamic complications and mucosal bleeding may increase, which is highly undesirable. Therefore, nasogastric tube placement may become a complex technique that requires significantly higher skill and the ability to quickly adapt if there are complications.

Many techniques have been suggested in the past to overcome the difficulty of conventional techniques such as head flexion [1,2], lateral rotation of the neck [2,3], reverse Sellick’s maneuver [2,4]; the use of split endotracheal tube [5], a GlideScope® (Verathon Medical, Bothell, WA, USA) [6,7], Magill forceps [8], oesophageal guide wire [9], the stylet [10], and frozen nasogastric tube [11]. However, the existence of various literature on techniques with different reported success rates shows that the search for the best method is still going on. The posterior tracheal wall has no cartilaginous support. It is only supported by a thin band of smooth trachealis muscle. The trachealis muscle is present on the posterior aspect of the tracheal wall and is next to the anterior esophagus [12]. The inflated cuff of the endotracheal tube puts pressure on the esophagus and compresses the esophagus posteriorly through the membranous portion of the trachea, thus limiting the space for the passage of the nasogastric tube. Therefore, we hypothesize that deflating the endotracheal tube cuff will facilitate easy passage of the nasogastric tube. However, the search of available literature did not bring any study evaluating the role of endotracheal tube cuff deflation in facilitating the passage of nasogastric tubes in anesthetized and intubated patients. Hence, the present study was conducted to assess the effect of deflating endotracheal tube cuff in facilitating nasogastric tube placement in an anesthetized and intubated patient.

## Materials and methods

Study design

After receiving approval from the Biomedical Research Ethics Committee, Pandit Bhagwat Dayal Sharma Post Graduate Institute of Medical Sciences, Rohtak, India (approval number: BREC/21/162), the current prospective, randomized interventional study was carried out in the Department of Anesthesiology and Critical Care. This study is registered with the clinical trials registry of India (CTRI/2022/01/039285).

Study population

This study comprised 80 patients between the ages of 18 and 60 who were scheduled for elective abdominal procedures and had an American Society of Anaesthesiologists (ASA) physical status I or II, as well as Mallampatti Grade (MPG) I or II, requiring nasogastric tube insertion. Patients with a history of bleeding disorders, history of head and neck deformities, the base of skull fracture, anticipated difficult airway, radiotherapy to head and neck area, history of nasal deformity or obstruction, and patients with esophageal varices were excluded from the study.

Intervention

Patients were randomly assigned to two groups using a sealed opaque envelope approach after receiving informed and written consent. Group A followed the nasogastric tube insertion without endotracheal cuff deflation, and Group B adopted the nasogastric tube insertion with endotracheal tube cuff deflation. In both groups, lateral pressure was applied at the level of the cricoid ipsilateral to the nostril in which the nasogastric tube was inserted. This was done to prevent the impaction of the nasogastric tube in the piriform sinus as it obliterates the potential space.

Preanesthetic evaluation was done a day prior to surgery. A nasal patency test was done preoperatively to find the more patent nostril and rule out nasal deformities like a spur or deviated nasal septum. Patients were induced using an injection of fentanyl, propofol, and a non-depolarising neuromuscular blocking agent. All the patients underwent orotracheal intubation to secure the airway with an appropriate size tube. Sevoflurane and oxygen in nitrous oxide were used to maintain anesthesia (50:50).

After tracheal intubation, xylometazoline nasal drops were instilled into both nostrils. An anesthetist with a minimum of five years of experience did nasogastric tube insertion. In both groups, a sterile, well-lubricated 14 French ROMSONS (Agra, India) nasogastric tube was inserted via a more patent nostril until it reached a length of 10 cm. Then, lateral pressure was applied to the neck around the cricoid area on the side similar to the nostril in which a nasogastric tube was inserted in both groups. In Group A, nasogastric tube placement was done through the more patent nostril without deflating the endotracheal tube's cuff. In Group B, nasogastric tube placement was done through the more patent nostril after deflating the endotracheal tube's cuff. The endotracheal tube's cuff was inflated after the correct placement of the nasogastric tube was confirmed by auscultation with a stethoscope in the epigastrium. If the first attempt failed, the nasogastric tube was withdrawn, cleaned, re-lubricated, and inserted into the same nostril using the same technique previously used. The number of attempts was noted along with the overall success rate. It was considered a failed placement if more than two attempts were needed.

If more than two failed attempts occurred, the nasogastric tube was guided into the esophagus using Magill forceps under laryngoscope guidance as a rescue maneuver. The time was calculated from when the nasogastric tube was inserted through the nostril until it was successfully placed. Complications like kinking, coiling, or bleeding was noted in both groups.

The primary goal of this study was to see if there was a difference in the overall success rate of both approaches for inserting a nasogastric tube and the first-attempt success rate. The secondary goal was to compare the time required for the successful placement of the nasogastric tube, the incidence of mucosal bleeding, kinking, coiling, and any other complications.

Sample size calculation

A previous study by Mahajan et al. suggested a 50% incidence success rate using the conventional technique of nasogastric tube insertion [1]. Therefore, we assumed that a 30% increase in the success rate of nasogastric tube placement using the technique of endotracheal cuff deflation compared to the conventional technique would be clinically significant. With α=0.05 and an 80% power of research (1−β), a sample size of 38 cases was necessary for each group.

Statistical analysis

The data gathered was entered into a Microsoft Excel spreadsheet (Microsoft Corp., Redmond, WA, USA). The Statistical Package for the Social Sciences (SPSS) version 20.0 (IBM Corp., Armonk, NY, USA) was used to conduct all analyses. An unpaired t-test was used to examine age, body mass index (BMI), and nasogastric tube insertion time. In addition, ASA status, MPG grading, sex, number of attempts, successful insertion, and complications after nasogastric tube insertion were compared using the chi-square test. The statistical significance level was chosen at p=0.05.

## Results

**Figure 1:**
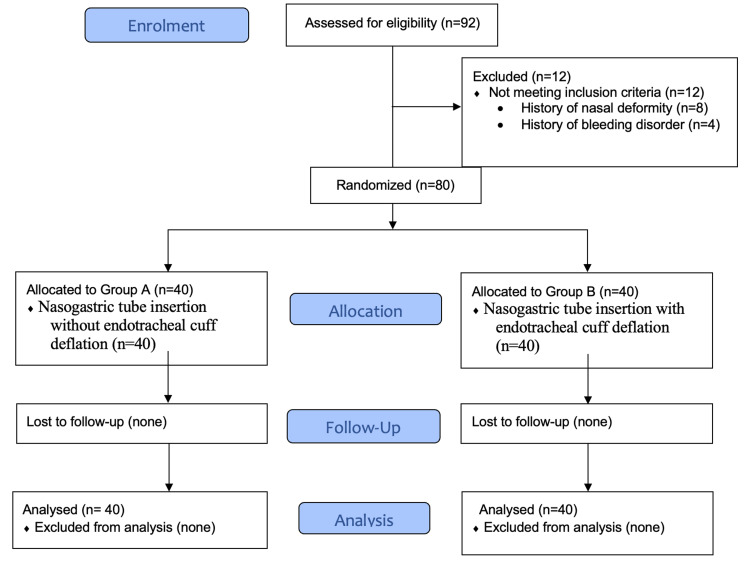
Study consort diagram

Our research comprised a total of 80 patients. The study's flow diagram is presented in (Figure 1). There were no differences in patient characteristics like mean age (p=0.36), sex (p=0.26), BMI (p=0.38), ASA status (p=0.81) and Mallampatti score (p=0.46) between the two groups (Table 1).

**Table 1:** Demographic parameters. Values expressed as mean and standard deviation, n=number (%) where appropriate. BMI: Body mass index, ASA: American Society of Anesthesiologists physical status

Parameter	GROUP A	GROUP B	p-value
Mean age (in years)	42.5 ± 7.133	44 ± 7.55	0.3639
Male	18 (45)	24 (60)	0.2629
Female	22 (55)	16 (40)	
BMI (kg/m^2^) (mean ± standard deviation)	15.53 ± 1.92	15.99 ± 3.759	0.3816
ASA I	12 (30)	14 (35)	0.8113
ASA II	28 (70)	26 (65)	
Mallampatti score I	10 (25)	14 (35)	0.4642
Mallampatti score II	30 (75)	26 (65)	

In Group A, the first-attempt nasogastric tube insertion success rate was 25%, whereas in Group B it was 55%. A second attempt was required in 30% of Group A cases and 25% of Group B cases (p=0.014). The overall success rate in Group B was significantly higher, at 80% vs 55% in Group A (p =0.016). The nasogastric tube was ultimately placed in the remaining cases in both groups using the rescue technique. The rescue technique was used in 18 (45%) patients in Group A and eight (20%) patients in Group B (p=0.0169). In Group A, the mean time for successful insertion was 65.4± 28.72 seconds compared to 43.5 ± 25.37 seconds in Group B (p=0.0005) (Table 2).

 

**Table 2:** Study parameters expressed as n=number (%). The time required is expressed as mean time and standard deviation.

Parameter	Group A	Group B	p-value
First-attempt insertion	10 (25)	22 (55)	0.014066
Second-attempt insertion	12 (30)	10 (25)	
Rescue maneuvers required	18 (45)	8 (20)	
Successful insertion	22 (55)	32 (80)	0.016984
Time required in seconds (mean ± standard deviation)	65.4 ± 28.725	43.5 ± 25.37	0.0005

The complications occurring during nasogastric tube insertion included kinking, coiling, and bleeding. In Group A, 12 patients (30%) had bleeding episodes, whereas in Group B only four patients (10%) had bleeding episodes. Group A had considerably more bleeding (p=0.025). In Group A, kinking of the nasogastric tube occurred in nine patients (22.5%), whereas in Group B eight patients (20%) encountered kinking. Coiling of the nasogastric tube occurred in 11 (27.5%) cases in Group A vs 4 (10%)cases in Group B. Incidence of complications like kinking and coiling was not statistically significant between the groups (p=0.784 and p=0.085, respectively) (Table 3). 

**Table 3:** Incidence of complications expressed as n=number (%)

Complications	Group A	Group B	p-value
Bleeding	12 (30)	4 (10)	0.02534
Kinking	9 (22.5)	8 (20)	0.7846
Coiling	11 (27.5)	4 (10)	0.08567

## Discussion

The insertion of a nasogastric tube in anesthetized and intubated patients might be complex due to the patient's inability to swallow. Anatomic considerations have been determined to be the most common cause of incorrect nasogastric tube placement [13]. The piriform sinus, the arytenoid cartilage, and the esophagus, which can be compressed by the endotracheal tube's inflated cuff, are the most typical sites of resistance [4,13].

Impaction of nasogastric tube in the piriform sinus and arytenoid cartilage may prevent its correct placement. This problem is significantly reduced by maneuvers like neck rotation and lateral neck pressure [2,3]. However, these maneuvers cause the ipsilateral piriform sinus to collapse and the arytenoid to shift medially [13].

The endotracheal tube's inflated cuff is another anatomical constraint that makes it challenging to navigate the nasogastric tube through the esophagus smoothly. The endotracheal tube's inflated cuff may compress the esophagus posteriorly through the membranous portion of the trachea and thus narrows down the space available for the passage of the nasogastric tube [2].

Various research on the role of lateral neck pressure has been conducted in the past. However, the literature still lacks evidence on the endotracheal tube cuff deflation technique. The results of our study revealed that endotracheal tube cuff deflation (Group B) significantly improved the first-attempt success rate of nasogastric tube passage compared to the group where the endotracheal tube cuff was kept inflated (Group A). In Group A, 25% of patients had first-attempt successful placement of nasogastric tube as compared to 55% in Group B. Overall success rate was also significantly high in Group B (80%) when compared to Group A (55%) with a p-value of 0.01 (Table 2). We applied lateral neck pressure in both groups to eliminate bias in readings.

Neck flexion and lateral neck rotation have been invariably used in various studies with a high success rate. For example, Siddhartha et al. (2017) found that lateral pressure and neck flexion improved the success rate of nasogastric tube placement in intubated patients undergoing laparoscopic hysterectomies [14]. Illias et al. (2013) also found that neck flexion and lateral neck pressure improved the overall success rate of correct nasogastric tube insertion as compared to the conventional method (88% vs 60%) [2]. However, all of these techniques involve manipulation of the cervical spine. This movement is not advisable for cervical spine trauma, spondylosis, herniated disc, and other disorders [15]. So inserting a nasogastric tube becomes challenging. This study did not involve any manipulation at the cervical spine level, so we recommend this technique in cases where neck mobility is not permissible.

The time required for successful nasogastric tube insertion in Group B was significantly less than in Group A (p=0.0005) (as seen above in Table 2). The time required for endotracheal tube cuff deflation is minimal, so this intervention did not increase the total time duration in this group. Moreover, the first attempt at a successful passage of the nasogastric tube was high in Group B, so the overall time duration was reduced. In Group A, the time required for rescue maneuvers was also added to the total time duration for successful placement.

In the present study, the incidence of complications like kinking and coiling was not statistically significant between the two groups (as seen above in Table 3). We believe that kinking and coiling of the nasogastric tube can occur due to anatomical resistance and was reduced by lateral neck pressure in both groups. In Group B, cuff deflation further helped to overcome the resistance. Kinking can also occur due to the properties of the nasogastric tube-like soft, pliable material and distal apertures. In this research, the same type of a 14-Fr nasogastric tube was utilized in both groups to eliminate bias caused by nasogastric tube physical features. The occurrence of bleeding was significantly high in Group A (p=0.023) (Table 3). This may be attributed to the fact that multiple insertion attempts and rescue techniques increase the potential of trauma to nasopharyngeal structures and increase the chances of bleeding. 

This study had certain limitations. First, the performing anesthetist was not blinded to the type of intervention used. Second, this study was limited to ASA status 1 or 2, with Mallampatti Grade 1 or 2. Third, radiological confirmation remains the gold standard, but we used the auscultation method to confirm correct placement. Fourth, deflating the endotracheal tube is of concern in patients with inadequate fasting and patients who are prone to aspiration like in pregnancy and severe obesity, etc. So this technique is of limited use in such scenarios. Fifth, this research did not consider various other factors such as the size of the tongue, thyromental distance, and the height of the head ring/pillow. Further studies designed to overcome the constraints mentioned above are recommended. It would remain future scope to conduct trials in patients with limited neck mobility and difficult airway.

We believe the endotracheal tube cuff deflation technique is a simple and feasible technique for nasogastric tube placement in anesthetized patients. It does not require any skill or instruments for nasogastric tube placement. This is the first study to evaluate the role of endotracheal tube cuff deflation in facilitating nasogastric tube placement, and we found favorable outcomes.

## Conclusions

The endotracheal tube cuff deflation-assisted technique improved the chances of successful nasogastric tube placement. It also helped to decrease complications like bleeding by avoiding multiple attempts. Therefore, we recommend using this innovative approach for nasogastric tube insertion in anesthetized and intubated patients.

## References

[1] Mahajan R, Gupta R, Sharma A (2005). Role of neck flexion in facilitating nasogastric tube insertion. Anesthesiology.

[2] Illias AM, Hui YL, Lin CC, Chang CJ, Yu HP (2013). A comparison of nasogastric tube insertion techniques without using other instruments in anesthetized and intubated patients. Ann Saudi Med.

[3] Bong CL, Macachor JD, Hwang NC (2004). Insertion of the nasogastric tube made easy. Anesthesiology.

[4] Parris WC (1989). Reverse Sellick maneuver. Anesth Analg.

[5] Dobson AP (2006). Nasogastric tube insertion—another technique. Anaesthesia.

[6] Wan Ibadullah WH, Yahya N, Ghazali SS, Kamaruzaman E, Yong LC, Dan A, Md Zain J (2016). Comparing insertion characteristics on nasogastric tube placement by using GlideScope™ visualization vs. MacIntosh laryngoscope assistance in anaesthetized and intubated patients. Braz J Anesthesiol.

[7] Kim HJ, Park SI, Cho SY, Cho MJ (2018). The GlideScope with modified Magill forceps facilitates nasogastric tube insertion in anesthetized patients: a randomized clinical study. J Int Med Res.

[8] Staar S, Biesler I, Müller D, Pförtner R, Mohr C, Groeben H (2013). Nasotracheal intubation with three indirect laryngoscopes assisted by standard or modified Magill forceps. Anaesthesia.

[9] Kirtania J, Ghose T, Garai D, Ray S (2012). Esophageal guidewire-assisted nasogastric tube insertion in anesthetized and intubated patients: a prospective randomized controlled study. Anesth Analg.

[10] Tsai YF, Luo CF, Illias A, Lin CC, Yu HP (2012). Nasogastric tube insertion in anesthetized and intubated patients: a new and reliable method. BMC Gastroenterol.

[11] Chun DH, Kim NY, Shin YS, Kim SH (2009). A randomized, clinical trial of frozen versus standard nasogastric tube placement. World J Surg.

[12] Furlow PW, Mathisen DJ (2018). Surgical anatomy of the trachea. Ann Cardiothorac Surg.

[13] Ozer S, Benumof JL (1999). Oro- and nasogastric tube passage in intubated patients: fiberoptic description of where they go at the laryngeal level and how to make them enter the esophagus. Anesthesiology.

[14] Siddhartha BS, Sharma NG, Kamble S, Shankaranarayana P (2017). Nasogastric tube insertion in anesthetized intubated patients undergoing laparoscopic hysterectomies: a comparative study of three techniques. Anesth Essays Res.

[15] Alizada M, Li RR, Hayatullah G (2018). Cervical instability in cervical spondylosis patients: significance of the radiographic index method for evaluation. Orthopade.

